# Identification of* Clostridium difficile* Asymptomatic Carriers in a Tertiary Care Hospital

**DOI:** 10.1155/2017/5450829

**Published:** 2017-10-02

**Authors:** André Luiz de Oliveira Silva, Alexandre R. Marra, Marinês Dalla Valle Martino, Ana Carolina Cintra Nunes Mafra, Michael B. Edmond, Oscar Fernando Pavao dos Santos

**Affiliations:** ^1^Clinical Laboratory, Hospital Israelita Albert Einstein, São Paulo, SP, Brazil; ^2^Division of Medical Practice, Hospital Israelita Albert Einstein, São Paulo, SP, Brazil; ^3^Office of Clinical Quality, Safety and Performance Improvement, University of Iowa Hospitals and Clinics, Iowa City, IA, USA; ^4^Instituto Israelita de Ensino e Pesquisa Albert Einstein, Hospital Israelita Albert Einstein, São Paulo, SP, Brazil

## Abstract

**Background:**

The diagnosis of* Clostridium difficile* infection (CDI) increases concern that asymptomatic carriers of toxigenic* C. difficile* may be diagnosed with CDI.

**Methods:**

A matched case control study was conducted in inpatients in a tertiary care center. The first 50 patients with diarrhea and a positive polymerase chain reaction (PCR) test beginning February 1, 2015, were identified as cases. Control patients were hospitalized patients receiving antibiotics, but with no diarrhea, housed in a room as close as possible to each case during the same admission time. A convenience sample of healthcare workers who cared for* C. difficile* infected patients was also tested.

**Results:**

We found two positive PCR results for* C. difficile* in controls (4.1%). None of these healthcare workers were positive for* C. difficile* by PCR. There was no difference between groups with respect to overall antibiotic use before the requested PCR for* Clostridium difficile* (*p* = 0.359). The majority of cases had a high proportion of gastrointestinal disorders (71.4%) compared with control (8.2%), *p* < 0.001. Patients with neoplasia had a higher chance of being identified as cases (*p* = 0.041).

**Conclusions:**

PCR should not be the only diagnostic tool but should be complementary to other methods and to the medical history.

## 1. Background

Over the past few decades,* Clostridium difficile* (CD) has become a dilemma for global public health. It is the main cause of healthcare-associated diarrhea [1] and is associated with antibiotic use. The estimated number of deaths annually from this infection in the US is 15,000–20,000 [2, 3], with mortality rates estimated at 6.9% within 30 days and 16.7% within a year of diagnosis [4].

Laboratory tests are essential for diagnosis, despite some conflicting results between types of tests [5]. Alone, a positive result from a lab test (ELISA, polymerase chain reaction [PCR] or stool culture) is not sufficient to define the diagnosis: a clinical assessment is the key to the interpretation and validation of diagnostic methods of CD infection [6].

For decades, tests to detect the toxin were favored over culture as the mainstay of diagnosis, since they provide more rapid turnaround times [7]. However, molecular tests that assess toxin genes, including PCR, are also rapid but more sensitive than toxin testing. Automated RT-PCR (real-time polymerase chain reaction) is the test used to detect the genes encoding toxin. The major factors determining* C. difficile* virulence are enterotoxin A and cytotoxin B. The genes encoding toxin A* (tcdA)* and toxin B* (tcdB)* are part of the pathogenicity locus (PaLoc).

The significance of a positive PCR result creates difficulties for clinical interpretation, due to the large number of positive tests from individuals without disease.* Clostridium difficile* colonization is 5- to 10-fold more common than symptomatic infection [8].

The main risk factors associated with* Clostridium difficile *diarrhea (CDD) include antibiotic use, older age, comorbidities, and prolonged hospital stay [9]. Many hospitalized patients receive antibiotic therapy, which favors development of infection with this agent.

In patients admitted to intensive care, colonization by toxin-producing* C. difficile* is an independent risk factor for the development of* Clostridium difficile* infection (CDI). However, further studies are necessary to identify populations with higher rates of colonization by toxigenic* Clostridium difficile* possibly benefiting from screening or avoidance of agents known to promote CDI [10].

After the incorporation of PCR testing into routine testing for diagnosis of* C. difficile* at our hospital, there was a significant increase in* C. difficile* cases causing great concern among physician and nurses. We noted that many patients were being treated for* C. difficile* that did not have diarrhea, and there were concerns that healthcare workers may become infected. Therefore, we sought to identify the presence of* C. difficile* in hospitalized patients receiving antibiotics without diarrhea, as well as in healthcare workers who cared for patients with* C. difficile* disease.

## 2. Materials and Methods

A matched case control study was conducted in inpatients at Hospital Israelita Albert Einstein (HIAE), a 670-bed facility in the city of São Paulo, SP, Brazil. This study was approved by the Institutional Review Board of Hospital Israelita Albert Einstein (IRB). Written informed consent was obtained from the patients or their legal representatives, as well as the healthcare workers that participated in this study.

The first 50 patients with diarrhea and a positive PCR test beginning February 1, 2015, were identified as cases. Laboratory diagnosis of CDI was established by the detection of sequences of the genes for toxin B* (tcdB)* and binary toxin* (cdt)* and by elimination of* tcdC* nt 117 (*tcd*CΔ117) by Cepheid Xpert ®, which is one of the NAAT (nucleic acid amplification test) methods.

One control patient was matched to each case. Control patients were hospitalized patients receiving antibiotics, but with no diarrhea, housed in a room as close as possible to each case during the same admission time. If two patients meeting the criteria were available at the same time, the first to have stool available for testing was included.

All hospital rooms are private, and each has dedicated noncritical devices for patient care (e.g., stethoscopes and thermometers). There are one sink (with a bottle of chlorhexidine) and one alcohol gel dispenser on the wall inside of each room and one alcohol gel dispenser between each room in the corridor.

Healthcare-associated infection (HAI) was defined as an episode of diarrhea >72 h after admission, pseudomembranous colitis or toxic megacolon after this period, or a positive test for CDI within 72 hours of discharge in the setting of diarrhea. Whenever CDI was suspected, the attending or on-duty physician ordered PCR for CD; samples were collected and sent to the microbiology lab according to the guidelines for sample stability. Asymptomatic carriers were defined as those with a positive* C. difficile* toxin test, but no diarrhea [10, 11].

Contact precautions were utilized empirically for all patients who developed diarrhea until the final result of the* C. difficile* PCR test was available. When the patient had documented* Clostridium difficile* diarrhea, contact precautions were maintained and caregivers were advised as per the hospital policy to use chlorhexidine for hand hygiene; however, no prompts, such as signs recommending the avoidance of alcohol hand rub, were used. Asymptomatic carriers were not placed in contact precautions. If the PCR results for* C. difficile* were positive in controls, they were not treated.

The following variables were obtained by search of medical records: demographics (age, gender) and epidemiological and clinical data (comorbidities and corresponding therapies, antibiotic therapy prior to* C. difficile* testing, invasive devices, and surgical procedures).

One healthcare worker, who cared for a* C. difficile* infected patient, was tested for each included case.

### 2.1. Statistical Analysis

Categorical variables were described by absolute and relative frequencies, and numerical variables were expressed by median and interquartile range (IQR), 1st and 3rd quartiles.

In the comparison between paired groups, McNemar's hypothesis tests were used for categorical variables and Wilcoxon's test for numerical variables. We matched the patients only on geographic proximity. Analyses for independent data are based on the assumption that the control sample was randomized to all nondiseased patients available in the study period, which is not the case in this study.

Analyses were performed using the R software, version 3.1.3 (R Core Team, 2015) [12]. The significance level adopted was 5%.

## 3. Results

**Figure 1:**
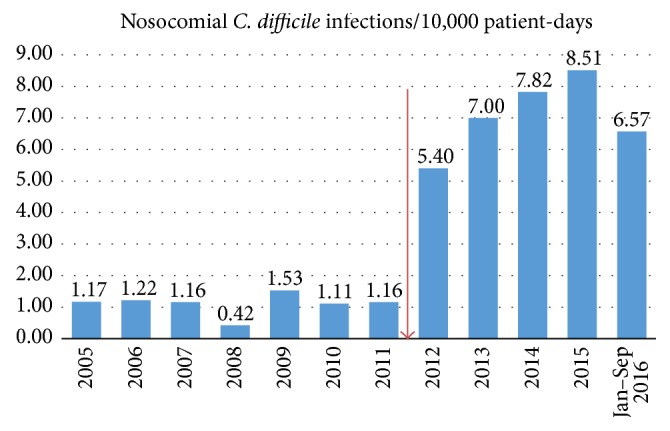
Incidence rate of nosocomial* C. difficile* infection. Red arrow: implementation of* C. difficile* PCR diagnostic testing.

From February 2015 through December 2015, we had 177,427 patient-days. During the same period, we found 335 cases with a positive PCR test for* C. difficile*. The diagnostic testing method for* C. difficile* was changed from ELISA to PCR in 2012 and following that the number of cases annually increased by 5- to 8-fold (Figure 1).

We found two positive PCR results for* C. difficile* in controls (4.1%). We tested 49 healthcare workers who cared for* C. difficile* patients during the study period. None of these healthcare workers were positive for* C. difficile* by PCR.

Risk factors for cases and controls are shown in Tables 1 and 2. One case was captured twice (it was collected by mistake); therefore, the sample was reduced to 49 matched pairs when we performed the analysis.

Table 1 refers to the numeric variables that characterize cases and controls, that is, age, number of comorbidities, number of antibiotics used (before the PCR for* C. difficile* being requested), and antibiotic use (before the PCR for* C. difficile* being requested) in days. The number of antibiotics used (prior to* C. difficile* testing) was not significantly different between the two groups (*p* = 0.058), with the overall median being 1 drug (IQR: 1-2). The likelihood of a positive result increased with assessed variables.

Table 2 shows that 80.6% of the cases received antibiotics, predominantly cephalosporins (30.6%); 74.4% received antibiotics during the index hospitalization, whereas 25.6% received antimicrobial treatment in the week before hospital admission.

Table 2 also shows that there was no difference between groups with respect to overall antibiotic use before the requested PCR for* Clostridium difficile* (*p* = 0.359) and gives comparative details. Significant differences were found in the use of quinolones (*p* = 0.008), with more frequent use in controls (in 18.4% of the pairs, only the control patient had used this drug), and in intravenous glycopeptides (*p* = 0.004), with more frequent use in cases (in 34.7% of the pairs, only the case had been treated with this class of drugs). Half of the cases had an antibiotic before hospitalization, whereas all the controls initiated the antibiotic after admission (*p* = 0.001).

Table 3 shows the importance of devices, such as catheters, present in 95.9% of the cases. Most subjects (99%) had no prior hospitalization; 43.9% experienced gastrointestinal disorders (e.g., abdominal pain and abdominal distention) during the index hospitalization, indicating that nearly half of these subjects had clinical manifestations of CDI. The majority of cases had a high proportion of gastrointestinal disorders (71.4%) compared with control (8.2%), *p* < 0.001. Patients with neoplasia had a higher chance of being cases (*p* = 0.041).

## 4. Discussion

Our study demonstrated an increase in the number of* C. difficile* cases after implementing the PCR method. In our small sample of the studied patients receiving antibiotics but without diarrhea, the prevalence of* C. difficile* colonization was less than 5%. The only nonantimicrobial predictor for CDI was gastrointestinal symptoms (*p* < 0.001). This is an important point to consider when clinicians make the decision to request a* C. difficile* diagnostic test. We did not detect any positive* C. difficile* results by PCR in the healthcare workers that took care of* C. difficile* patients. Recently, it has been noted that advances in clinical microbiology have led to more sensitive tests that may falsely increase HAI rates and not result in safer care [13].

The diagnosis of* C. difficile* is usually based on clinical manifestations of diarrhea and the detection of A- or B-toxin-producing genes. However, the labor-intensive cell-mediated cytotoxicity assay is considered the gold standard due to its high specificity [11, 14]. Several rapid immunoenzymatic assays have been developed to detect these genes. However, these tests have lower sensitivity and specificity when compared to the cell-mediated cytotoxicity assay. PCR-based methods have been developed to detect genes for toxins A and/or B, with high sensitivity compared to cell-mediated cytotoxicity and immunological assays [15].

The pathogenesis of CDI differs from other illnesses since one or more risk factors are generally required to trigger the disease. Most patients have no clinical manifestations and do not require treatment. These are considered asymptomatic carriers [16] and may represent 7–26% of adult inpatients [17]. These patients represent a potentially important source of transmission of the agent to other patients [18].

Our diagnostic method was PCR. Molecular tests are more sensitive than other methods to detect* C. difficile* in the stools [7]. Hospitals report a 50%–100% increase in the rate of CDI when toxin tests are replaced with molecular tests [19, 20].

Another study assessed inpatients with symptoms lasting more than 72 hours, using liquid stool samples. For each patient, only the first sample was tested [21] among adult inpatients with suspect CDI; nearly all deaths and complications of CDI occurred in those patients with a positive immunoassay for toxin. Patients with a positive molecular test and a negative toxin immunoassay had comparable results to patients with no detection of* C. difficile* by whichever method. The use of molecular tests alone to diagnose CDI, without the toxin or host response tests, will likely lead to an excessive number of positively diagnosed cases, excessive treatment, and increased healthcare costs [21]. Most patients with a negative test for* C. difficile* toxin do not require specific treatment, even though there may be a rationale for identifying carriers and, thus, preventing transmission [22].

In another study, nearly half of the patients who responded to treatment with symptom relief continued to eliminate spores for up to 6 weeks. Even after a successful treatment, stool tests quite often remain positive for* C. difficile* for several months, suggesting persistent colonization after clinical illness. Therefore, repeated stool tests using PCR, either to check for positivity or as a test of cure, are not recommended [17]; the correlation between the clinical condition and lab results should guide management.

The effect of aging relates to the increased rate of chronic diseases and the presence of comorbidities. Many hospitalizations are due to illnesses requiring the use of antibiotics, and the association of more than two drugs and the duration of use are shown to have a role in the incidence of* C. difficile* diarrhea. These factors are related to changes in the gastrointestinal microbiota.

The limitations of our study include the exclusive use of molecular tests, not accompanied by toxin detection tests. In addition, our sample was small, due to the costs of PCR, and the study was conducted at a single site; its results cannot be extrapolated to other healthcare institutions. Using a single episode of diarrhea to define cases likely included some patients that were colonized but having diarrhea due to other causes.

Our laboratory began to restrict testing to patients with three or more unformed stools within 24 hours in November 2016. It is likely that many asymptomatic carriers of toxigenic* C. difficile* with unformed stool due to other causes were diagnosed with CDI, resulting in unnecessary treatment and inflation of CDI rates [23, 24].

In conclusion, we recommend assessing patients for diarrhea and interpreting laboratory results considering the clinical setting and the likelihood of other etiologies for diarrhea in patients with a positive* C. difficile* test. PCR should not be the only diagnostic tool but should be complementary to other methods and to the medical history.

## Figures and Tables

**Table 1:** Description of numeric variables, continuous or discrete, by median, 1st quartile, 3rd quartile, and comparisons between the two groups of patients (*N* = 49 pairs).

Variable	All patients	Cases	Controls	*p*
	Median (*Q*1, *Q*3)	Median (*Q*1, Q3)	Median (*Q*1, *Q*3)	
Age (years)	68 (43, 78)	70 (46, 80)	66 (40, 77)	0.451
Number of comorbidities	1 (1, 2)	2 (1, 3)	1 (0, 2)	0.195
Number of antibiotics use before the requested PCR for *C. difficile*	1 (1, 2)	1 (1, 2)	1 (1, 2)	0.058
Duration of antibiotic use before the requested PCR for *C. difficile* (days)	4 (1, 8)	3 (1, 6)	5 (1, 8)	0.304

*Q*1: 1st quartile; *Q*3: 3rd quartile; *p* values were obtained by Wilcoxon's test.

**Table 2:** Antibiotic use in cases and controls.

Factors	Total (*N* = 98)	Cases	Controls	Odds ratio (95% CI)^*∗*^	*p*
Antibiotic use before *Clostridium difficile *testing	79 (80.6)	42 (85.7)	37 (75.5)	1.71 (0.62, 5.14)	(0.359)
Clindamycin	4 (4.1)	2 (4.1)	2 (4.1)	1.00 (0.07, 13.80)	>0.999
Metronidazole	8 (8.2)	5 (10.2)	3 (6.1)	1.67 (0.32, 10.73)	(0.724)
Polymyxin	2 (2.0)	2 (4.1)	0 (0.0)	—	(0.480)
Trimethoprim-sulfamethoxazole	7 (7.1)	3 (6.1)	4 (8.2)	0.67 (0.06, 5.82)	>0.999
Penicillins	7 (7.1)	5 (10.2)	2 (4.1)	2.50 (0.41, 26.25)	0.450
Cephalosporins	30 (30.6)	14 (28.6)	16 (32.7)	0.80 (0.27, 2.25)	0.814
Fluoroquinolones	13 (13.3)	2 (4.1)	11 (22.4)	0.01 (0.00, 0.51)	0.008
Carbapenems	17 (17.3)	11 (22.4)	6 (12.2)	2.25 (0.63, 10.0)	0.267
Aminoglycosides	3 (3.1)	3 (6.1)	0 (0.0)	—	0.248
Macrolides	9 (9.2)	4 (8.2)	5 (10.2)	0.67 (0.06, 5.82)	>0.999
IV glycopeptides	20 (20.4)	17 (34.7)	3 (6.1)	5.67 (1.64, 30.18)	0.004
Period of antibiotic use				—	0.001
Current admission	58 (74.4)	22 (52.4)	36 (100.0)		
One week prior to admission	20 (25.6)	20 (47.6)	0 (0.0)		

IV = intravenous. ^*∗*^Pair matched odds ratio with 95% CI [confidence interval] (impossible to calculate where there are no controls exposed, signaled by “—”). *p* values were obtained by McNemar's test.

**Table 3:** Nonantimicrobial predictors of *C. difficile* infection. Description of discrete variables by *n* (%) and comparisons between the two groups of patients (*N* = 49 pairs).

Factors	Total (*N* = 98)	Cases (*n* = 49)	Controls (*n* = 49)	Odds ratio (95% CI)^*∗*^	*p*
Urinary catheter (yes)	94 (95.9)	45 (91.8)	49 (100.0)	0.01 (0.00, 1.51)	0.134
Peripheral IV (yes)	59 (60.2)	26 (53.1)	33 (67.3)	0.46 (0.14, 1.30)	0.169
Central venous catheter (yes)	32 (32.7)	18 (36.7)	14 (28.6)	1.57 (0.56, 4.78)	0.480
Nasoenteric tube (yes)	2 (2.0)	2 (4.1)	0 (0.0)	—	0.480
Tube feeding (yes)	16 (16.3)	8 (16.3)	8 (16.3)	1.00 (0.23, 4.34)	>0.999
Surgical procedures (yes)	15 (15.3)	9 (18.4)	6 (12.2)	1.60 (0.46, 6.22)	0.579
Mechanical ventilation (yes)	3 (3.1)	3 (6.1)	0 (0.0)	—	0.248
Neoplasia (yes)	6 (6.1)	6 (12.2)	0 (0.0)	—	0.041
Gastrointestinal symptoms during hospitalization (yes)	43 (43.9)	39 (79.6)	4 (8.2)	—	<0.001
Patient origin				—	>0.999
Home	97 (99.0)	48 (98.0)	49 (100.0)		
Other	1 (1.0)	1 (2.0)	0 (0.0)		

^*∗*^Matched odds ratio with 95% CI [confidence interval] (impossible to calculate where there are no controls exposed, signaled by “—”). *p* values were obtained by McNemar's test.

## Additional Points

*Availability of Supporting Data*. The data set supporting the results of this article is included within the article.

## References

[1] Voth D. E., Ballard J. D. (2005). *Clostridium difficile* toxins: mechanism of action and role in disease. *Clinical Microbiology Reviews*.

[2] Klevens R. M., Edwards J. R., Richards C. L. (2007). Estimating health care-associated infections and deaths in U.S. Hospitals, 2002. *Public Health Reports*.

[3] Dallal R. M., Harbrecht B. G., Boujoukas A. J. (2002). Fulminant *Clostridium difficile*: an underappreciated and increasing cause of death and complications. *Annals of Surgery*.

[4] Dubberke E. R., Carling P., Carrico R. (2014). Strategies to Prevent *Clostridium difficile* Infections in Acute Care Hospitals: 2014 Update. *Infection Control & Hospital Epidemiology*.

[5] Goldenberg S. D., French G. L. (2011). Diagnostic testing for *Clostridium difficile*: a comprehensive survey of laboratories in England. *Journal of Hospital Infection*.

[6] Planche T., Wilcox M. (2011). Reference assays for *Clostridium difficile* infection: one or two gold standards?. *Journal of Clinical Pathology*.

[7] Burnham C.-A. D., Carroll K. C. (2013). Diagnosis of *clostridium difficile* infection: an ongoing conundrum for clinicians and for clinical laboratories. *Clinical Microbiology Reviews*.

[8] Polage C. R., Solnick J. V., Cohen S. H. (2012). Nosocomial diarrhea: evaluation and treatment of causes other than *Clostridium difficile*. *Clinical Infectious Diseases*.

[9] Silva M., Marra A. R., Camargo T. Z. S. (2013). Secular trends in the epidemiology of *Clostridium difficile* infection (CDI): relationship with alcohol gel and antimicrobial usage in a hospital. *International Journal of Infectious Diseases*.

[10] Kelly S. G., Yarrington M., Zembower T. R. (2016). Inappropriate *Clostridium difficile* Testing and Consequent Overtreatment and Inaccurate Publicly Reported Metrics. *Infection Control and Hospital Epidemiology*.

[11] Wilkins T. D., Lyerly D. M. (2003). *Clostridium difficile* testing: after 20 years, still challenging. *Journal of Clinical Microbiology*.

[12] Core Team R. (2015). *R, A language and environment for statistical computing*.

[13] Diekema D. J. (2017). Rising stakes for health care-associated infection prevention: implications for the clinical microbiology laboratory. *Journal of Clinical Microbiology*.

[14] Delmée M. (2001). Laboratory diagnosis of *Clostridium difficile* disease. *Clinical Microbiology and Infection*.

[15] Poutanen S. M., Simor A. E. (2004). *Clostridium difficile*-associated diarrhea in adults. *Canadian Medical Association Journal*.

[16] Baker I., Leeming J. P., Reynolds R., Ibrahim I., Darley E. (2013). Clinical relevance of a positive molecular test in the diagnosis of *Clostridium difficile* infection. *Journal of Hospital Infection*.

[17] Cohen S. H., Gerding D. N., Johnson S. (2010). Clinical practice guidelines for *Clostridium difficile* infection in adults: 2010 update by the society for healthcare epidemiology of America (SHEA) and the infectious diseases society of America (IDSA). *Infection Control and Hospital Epidemiology*.

[18] Riggs M. M., Sethi A. K., Zabarsky T. F., Eckstein E. C., Jump R. L. P., Donskey C. J. (2007). Asymptomatic carriers are a potential source for transmission of epidemic and nonepidemic *Clostridium difficile* strains among long-term care facility residents. *Clinical Infectious Diseases*.

[19] Koo H. L., Van J. N., Zhao M. (2014). Real-time polymerase chain reaction detection of asymptomatic *Clostridium difficile* colonization and rising *C. difficile*-associated disease rates. *Infection Control and Hospital Epidemiology*.

[20] Gould C. V., Edwards J. R., Cohen J. (2013). Effect of nucleic acid amplification testing on population-based incidence rates of *clostridium difficile* infection. *Clinical Infectious Diseases*.

[21] Polage C. R., Gyorke C. E., Kennedy M. A. (2015). Overdiagnosis of *clostridium difficile* infection in the molecular test era. *JAMA Internal Medicine*.

[22] Planche T. D., Davies K. A., Coen P. G. (2013). Differences in outcome according to *Clostridium difficile* testing method: a prospective multicentre diagnostic validation study of C difficile infection. *The Lancet Infectious Diseases*.

[23] Prior A.-R., Fitzpatrick F. (2015). *Clostridium difficile*—to test or not to test? Response to Kundrapu et al. *Infection Control and Hospital Epidemiology*.

[24] Kundrapu S., Sunkesula V., Tomas M., Donskey C. J. (2015). Response to prior and fitzpatrick. *Infection Control and Hospital Epidemiology*.

